# Rational design of hybrid DNA–RNA triplex structures as modulators of transcriptional activity *in vitro*

**DOI:** 10.1093/nar/gkac1131

**Published:** 2022-12-20

**Authors:** Alessandro Cecconello, Massimiliano Magro, Fabio Vianello, Friedrich C Simmel

**Affiliations:** Physik Department, Technische Universität München, Garching bei München 85748, Germany; Department of Comparative Biomedicine and Food Science, University of Padova, Legnaro 35020, Italy; Department of Comparative Biomedicine and Food Science, University of Padova, Legnaro 35020, Italy; Department of Comparative Biomedicine and Food Science, University of Padova, Legnaro 35020, Italy; Physik Department, Technische Universität München, Garching bei München 85748, Germany

## Abstract

Triplex nanostructures can be formed *in vitro* in the promoter region of DNA templates, and it is commonly accepted that these assemblies inhibit the transcription of the downstream genes. Herein, a proof of concept highlighting the possibility of the up- or downregulation of RNA transcription is presented. Hybrid DNA–RNA triplex nanostructures were rationally designed to produce bacterial transcription units with switchable promoters. The rate of RNA production was measured using the signal of a transcribed fluorescent RNA aptamer (i.e. Broccoli). Indeed, several designed bacterial promoters showed the ability of induced transcriptional inhibition, while other properly tailored sequences demonstrated switchable enhancement of transcriptional activity, representing an unprecedented feature to date. The use of RNA-regulated transcription units and fluorescent RNA aptamers as readouts will allow the realization of biocomputation circuits characterized by a strongly reduced set of components. Triplex forming RNA oligonucleotides are proposed as smart tools for transcriptional modulation and represent an alternative to current methods for producing logic gates using protein-based components.

## Graphical Abstract

**Graphical Abstract:**
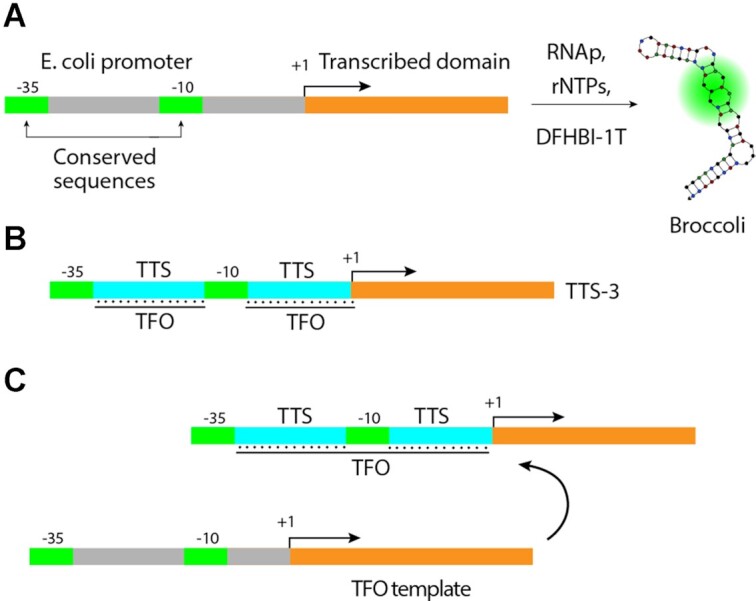
Different hybrid DNA–RNA triplex nanostructures regulate transcription of fluorescent RNA Broccoli (A), while forming structures between a dsDNA triplex–target site (TTS) and triplex forming oligonucleotide ssRNA (TFO), (B, C).

## INTRODUCTION

**Figure 1.**
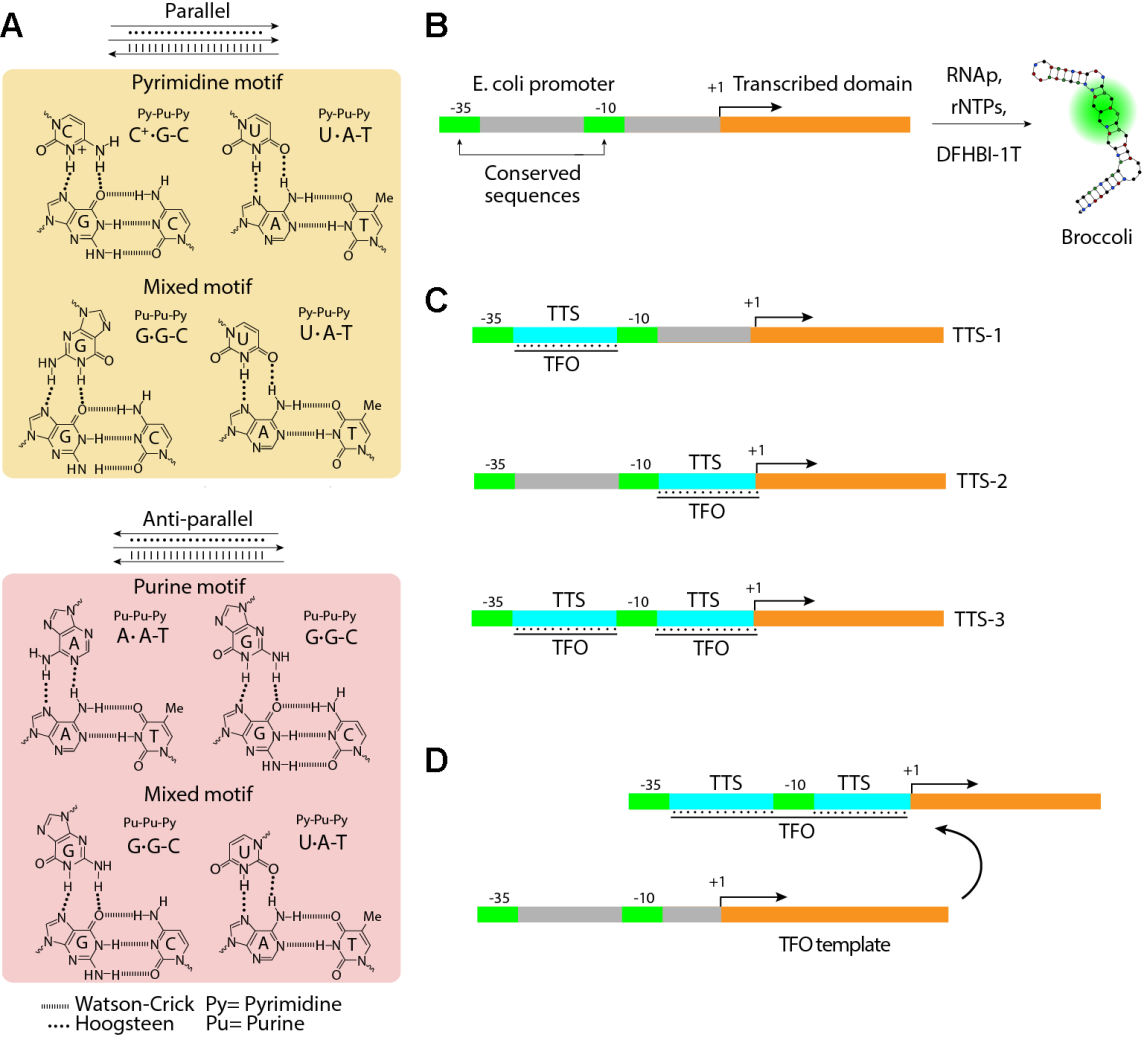
(**A**) DNA–RNA triplex motifs. Pyrimidine or purine motifs form preferentially parallel or antiparallel triplexes, respectively, while the mixed motif can in principle form a parallel triplex (upper part of the panel, with yellow background) or an anti-parallel triplex (lower part of the panel, pink background), therefore the nucleotide (i.e. G or U) comprised within the third filament of RNA in the triplex is depicted with mirrored geometries in the parallel and anti-parallel motifs. (**B**) *E. coli* transcription unit general architecture; (**C**) differently engineered dsDNA constructs containing one or two TTSs for triplex formation; and (**D**) *in situ* triplex formation in the presence of a TFO transcription unit and a TTS-containing transcription unit.

Beyond the Watson-Crick base-pairing of nucleic acids, DNA and RNA possess additional structural motifs such as triplexes and quadruplexes. Specifically, hybrid DNA and RNA triplex oligonucleotide nanostructures have been previously demonstrated to form in vitro between a double-stranded (ds) helical structure and a single-stranded (ss) oligonucleotide (1–4). The triplex forming oligonucleotide (TFO) binds at the major groove of the DNA double helix to the triplex target site (TTS), and interacts via hydrogen bonds with a polypurine (A and G) sequence (2,3). The association of a homopurine sequence in the dsDNA and the TFO, named Hoogsteen base-pairing, stabilizes the triplex structure. Four motifs are possible and they are characterized by TFO composition and orientation with respect to the polypurine sequence: antiparallel purine motif (containing A and G), parallel pyrimidine motif (containing C and U), and mixed motif (containing G and U) in either parallel or antiparallel orientation, Figure 1A. Triplex nanostructures were successfully introduced in nucleic acid-based nanotechnology designs *in vitro*, expanding the field toolbox of switchable mechanisms for sensing, smart drug delivery, and dynamic nanostructures control (5–8). Although triplex structures have been suggested to play a role in several biological mechanisms *in vivo* (9–15), to produce a complete picture and to identify specific triplex-forming RNAs, there is still a substantial need for experimental evidence of their activity. Recently, there has been growing interest in studying long noncoding RNAs (lncRNAs), a class of RNAs with several regulatory functions, in view of their involvement in triplex formation *in vivo* and its effect on gene expression (14,16–19). Furthermore, bioinformatic genomic sequence analyses have revealed that polypurine sequences are frequent in regulatory regions and in morphogenesis-related genes in eukaryotic organisms (20,21). While, on the one hand, these findings prompted the development of specific bioinformatic tools that look for triplex target sequences in genomes (20,22,23), on the other hand, they triggered the idea of using nucleic acid triplex structures as an interesting strategy to control gene expression *in vitro*, which may eventually also find applications *in vivo*.

Gene transcription in bacteria is regulated at the promoter region of a transcription unit mostly by its interaction with proteins. The RNA transcription complex is formed between the RNA polymerase (RNAp) core enzyme, one sigma factor, and their cognate DNA sequences (24,25). It has been reported that triplex structures at the promoter can inhibit transcription via competition between the formation of the transcription complex and the triplex structure. In addition, other natural or artificial triplex structures were reported to affect gene expression both *in vitro* and *in vivo* (11,12,26,27).

This phenomenon could be used, in principle, to generate artificial transcription circuits wired by oligonucleotides that form triplexes and, specifically, hybrid RNA–DNA triplexes. In fact, pioneering attempts to generate *in vitro* transcriptional circuits controlled by diffusing oligonucleotides were made previously, which were based on the completion or disruption of a nicked T7 promoter by toehold-mediated strand-displacement (so-called ‘genelets’) (28). By contrast, the use of triplexes does not require nicked DNA substrates and could operate with double-stranded DNA, which are less prone to degradation under biological conditions.

In this report, an artificial transcriptional regulation system was developed, in which bacterial promoters were designed to contain one or two TTSs for ribonucleic TFOs. The sequences were developed starting from the general *Escherichia coli* promoter architecture, Figure 1B, comprising two conserved domains (i.e. –35 and –10) that interact with sigma factors, separated by a non-conserved region (24). For the quantification of the promoter activity, we utilized the rate of transcription of the fluorescent RNA aptamer Broccoli (29). The designed promoters were modulated by the formation of RNA–DNA triplex structures in different positions at the promoter site, Figure 1C, while wiring was obtained by using two transcription units, Figure 1D.

## MATERIALS AND METHODS

All DNA and RNA sequences were ordered from IDT (Integrated DNA Technologies, USA) as freeze-dried material. Four synthetic ssRNA strands were used: TFO1 5’–UCCUCCUCUUCUCCU-3’, TFO2 5’-UGGUGGUGUUGUGGU-3’, TFO3 5’-UGGUGUUGUGGUGGU-3’ and TFO4 5’-AGGAGAAGAGGAGGA-3’. Each TFO was designed to form a triplex with a specific geometry with its respective target. Simply put, the target duplex should contain the exact same sequence as the TFO in the parallel or antiparallel orientation (except T is replacing U). All DNA sequences are detailed in Supplementary Table S1. Solutions were prepared in ultrapure water (resistivity 18.0 MOhm · cm) using a Sartorius Arium Pro Ultrapure Lab Water System. KCl, NaCl, Tris-acetate-EDTA (TAE), *N*,*N*,*N*’,*N*’-tetramethylethylenediamine (TEMED), and Tris were purchased from ROTH. Magnesium acetate was purchased from Fluka. SybrGold nucleic acid stain, Triton X-100 and ammonium persulfate were purchased from ThermoScientific. Acrylamide/bis-acrylamide solution was purchased from Sigma Aldrich/Merck. Broccoli fluorescent ligand (*Z*)-4-(3,5-difluoro-4-hydroxybenzylidene)-2-methyl-1-(2,2,2-trifluoroethyl)-1H-imidazol-5(4H)-one (DFHBI-1T) was purchased from Lucerna. σ70-saturated *E. coli* RNA polymerase (holoenzyme) was purchased from New England Biolabs (NEB). Dithiothreitol was purchased from Biochemica.

Double-stranded DNA 10 μM stock solutions were prepared in 50 mM KCl using a Mastercycler Nexus-GX from Eppendorf. All RNA polymerization experiments were carried out at 30°C in the recommended NEB buffer (40 mM Tris–HCl, 150 mM KCl, 10 mM MgCl_2_, 1 mM DTT, 0.01% Triton X-100) supplemented with additional MgCl_2_ to a final concentration of 30 mM. The additional magnesium was needed to ensure the formation of Broccoli and the triplexes. A Clariostar plate reader from BMG labtech with samples loaded in 384-well plates from Corning was used to acquire the fluorescence data. A JascoV-750, equipped with a Peltier unit, was used to perform temperature-dependent denaturation experiments to determine melting temperatures of the triplex structures. Melting temperature experiments were carried out in the same Mg-supplemented NEB buffer used in all other experiments. Triplex structures for EMSA (electrophoretic migration shift assay) experiments were prepared in the following way: First, the duplex DNA TTS was prepared using a 4-h ramp between 90°C and 4°C, then the specific TFO was added using the same Mg-modified NEB buffer used in all other experiments. Electrophoretic runs in 12% polyacrylamide, supplemented with 10 mM Mg^2+^, were performed using standard electrophoresis equipment (50 V for 3 h at 10°C) and stained with SybrGold.

## RESULTS

### Sequence-dependent stability of the triplex structures

**Figure 2.**
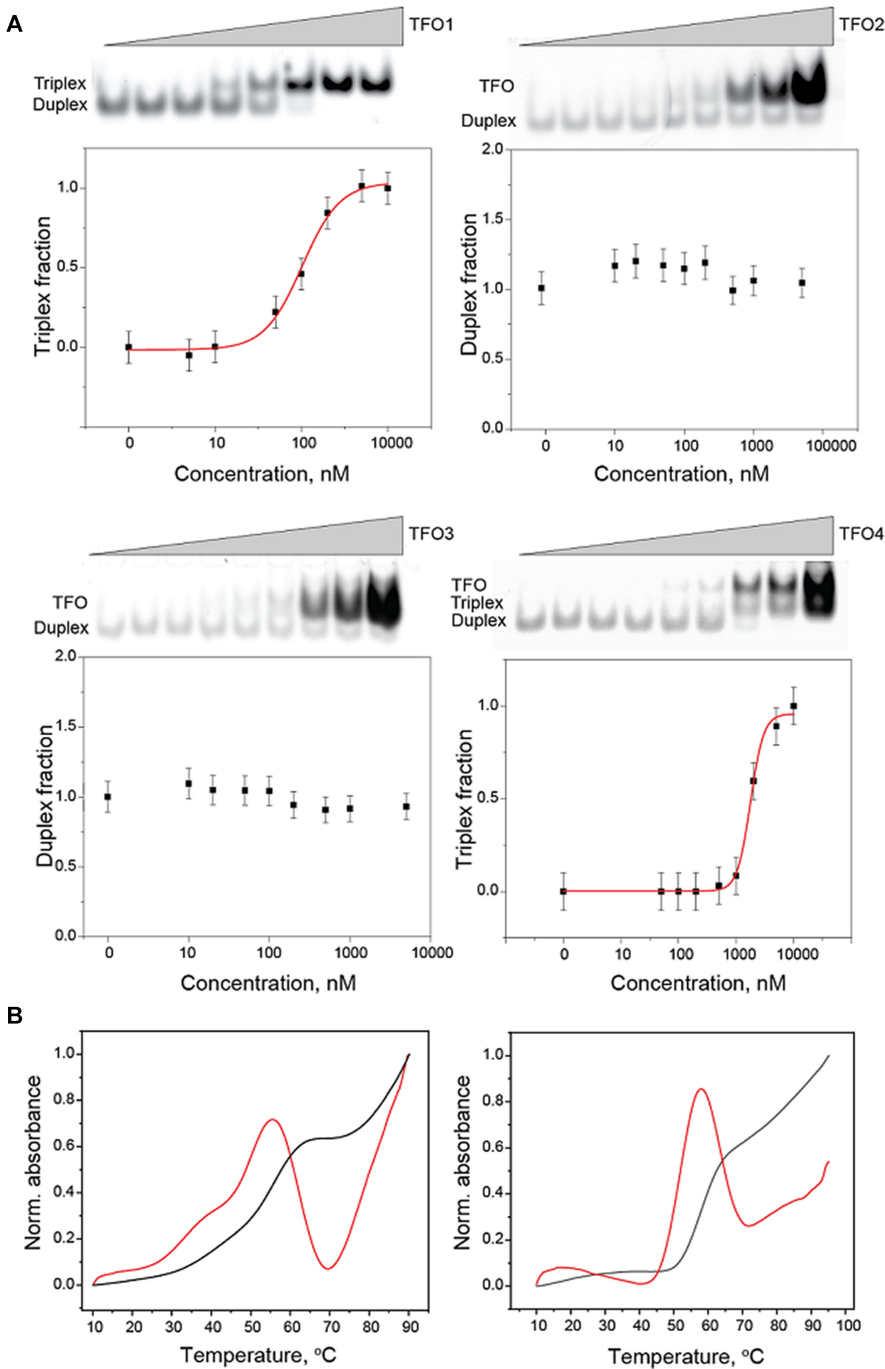
Stability of the different triplex motifs. (**A**) EMSA analysis of the duplex DNA TTS incubated with TFOs 1, 2, 3, and 4 in the concentration interval 0–10 μM or 0–100 μM, as indicated by the labels. (**B**) Melting curve analysis plots associated with triplexes TFO1 + TTS and TFO4 + TTS, left and right panels, respectively.

To be able to attribute a putative transcriptional modulation effect to triplex formation, the realization of hybrid triplexes at the TTS of the different promoters was first assessed using an electrophoretic mobility shift assay (EMSA), which was performed via polyacrylamide gel electrophoresis (PAGE) and temperature-dependent denaturation analysis (i.e. melting temperature determination). The full-length transcription units were re-designed and reduced to 15-base pair dsDNA constructs containing the TTS without adjacent sequences. The corresponding RNA TFOs 1, 2, 3, and 4 were designed to generate triplex structures at the TTS and form Hoogsteen interactions with the polypurine sequence in the parallel pyrimidine motif, the antiparallel purine motif, and the parallel/antiparallel mixed motif, respectively (for a list of DNA sequences, see Supplementary data, Table S1). Figure 2A shows the results of the EMSA analysis for the 15-bp TTS in the presence of different concentrations of TFO1, 2, 3, or 4, respectively. The band corresponding to the dsDNA TTS disappears at increasing concentrations of the TFO, while a more slowly migrating band corresponding to the triplex becomes visible at high TFO concentrations. No triplex formation was observed in the EMSA analysis for the parallel/anti-parallel mixed motifs. Specifically, the intensity of the band associated with the TTS did not change in the presence of different concentrations of TFOs 2 and 3, indicating negligible triplex formation within the range of the experimental TFO concentrations. Most importantly, the dissociation constants for the pyrimidine and purine motif triplexes were calculated to be 100 ± 1 nM and 1.8 ± 0.2 μM for TFO1 and 4, respectively. Although the different stabilities of purine and pyrimidine triplexes were already reported, there is still limited knowledge on the mechanisms stabilizing such hybrid structures, especially *in vivo*. In particular, although pioneering studies could not observe the formation of stable PuPuPy triplets, more recent publications found proof that sequence composition affects strongly the stability of such geometries (14,21,30,31). In addition, it is worth mentioning that bands attributed to the TFO excess are evident for triplexes comprising TFO2, TFO3 and TFO4 while excess TFO1 was not observed. This was attributed to the different compositions of the ssRNA sequences. In fact, TFO1 did not contain any guanine as opposed to 53% guanine content of the other TFOs which is known to stabilize quadruplex structures. The presence of these quadruplexes could explain the low migration rates of the bands associated with the TFO. The higher stability of the TFO1-containing triplex was also apparent in melting curves, where the DNA duplex melting temperature was correctly measured to be 58 ± 2°C while the release of the TFO could be clearly observed as a peak at 44 ± 2°C in the derivative analysis (red line), Figure 2B, left panel. Conversely, TFO4 was not sufficiently stable to be identified in the curve, Figure 2B, right panel.

In comparison to TFO4, the higher stability of the complex generated by TTS and TFO1 suggests its higher potential as a tight-binding oligonucleotide to inhibit transcriptional activity. Therefore, a DNA construct was designed to contain the 15 base polypurine TTS (TTS-1) in the template strand of the promoter consensus sequence between positions –35 and –10 (termed P1). Similar as for protein-based transcription factors binding in the P1 region, the TFO is then expected to compete with the polymerase for binding to the duplex.

### *Broccoli* production controlled by promoters containing one TTS

**Figure 3.**
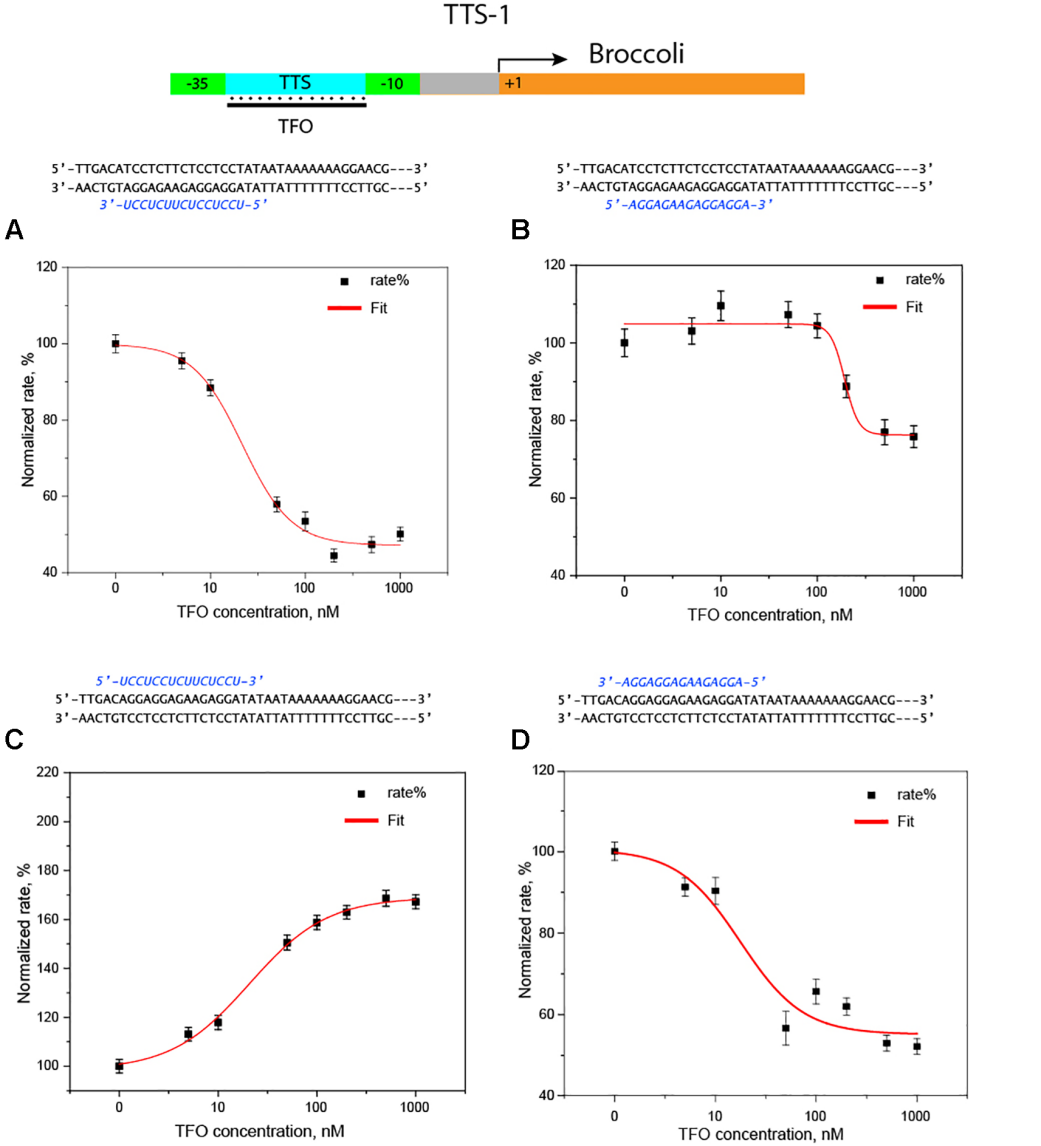
Analysis of the triplex formation effect on the production of Broccoli from transcription units engineered to contain TTS-1, as described in Figure 1C. (**A** and **B**) Kinetic analysis of the transcription units containing TTS-1, with the polypurine sequence in the template strand (P1), in the presence of different concentrations of TFOs 1 and 4, respectively. (**C** and **D**) Kinetic analysis of the transcription units containing TTS-1, with the polypurine sequence in the sense strand (P2), in the presence of different concentrations of TFOs1 and 4, respectively. The three dashes (—) indicate the same duplex sequence for all designs, namely, Broccoli template.

The synthetic promoters were tested in the presence of the *E. coli* RNAp saturated with σ70 factor (i.e. the protein interacting with the promoter conserved domains and the RNAp), for the rate of production of the Broccoli RNA aptamer (29) that forms a complex with the fluorescent dye DFHBI-1T, which has a significantly higher fluorescence than the free dye. Before adding the holoenzyme to the mixture, the transcription units were incubated for 30 minutes with different amounts of TFO (0 nM, 5 nM, 10 nM, 50 nM, 100 nM, 200 nM, 500 nM and 1 μM). During RNA polymerization, the fluorescence emission was monitored in a time interval of 12 hours and representative kinetics are reported in the SI, Supplementary Figure S1. The transcription rate was calculated by fitting linearly the time-dependent fluorescence intensity changes within an interval of 2 hours for all experiments. The resulting kinetic parameters are shown in TFO concentration-dependent plots, where polymerization rates were normalized to the unperturbed rate (i.e. in the absence of the respective TFO). The results for promoter P1 are shown in Figure 3, panels A and B. In agreement with the EMSA assay, the results indicate that the rate of Broccoli production is correlated with triplex formation and was significantly affected in the case of TFOs 1 and 4 while only a faint and erratic alteration in RNA transcription could be detected in the case of the mixed motif TFOs 2 and 3, Supplementary Figure S2. The TFO-dependent rates for TFOs 1 and 4 were fitted with a sigmoidal function and the calculated half-maximal effective concentrations (EC_50_) are reported in Table 1. Based on our preliminary assumptions, EC_50_ can be interpreted as a measure of the effectiveness of the TFO in inhibiting the transcription. In this view, the calculated EC_50_ values for TFOs 1 and 4, 22 ± 1 and 190 ± 2 nM, respectively, indicate that TFO1 is a stronger inhibitor than TFO4. Noteworthy, the EC_50_ values are fully in accord with the dissociation constants. Namely, the stronger the binding to the P1 promoter, the stronger is the effect of the oligonucleotide in hindering transcriptional activity.

**Table 1.** Kinetic parameters of the triplex systems comprising TTS-1, TTS-2, and TTS-3. and designed promoters P1, P2, P3 and P4

TTS	TTS position^a^	Triplex motif^b^	EC_50_ (nM)^c^	Modulation %^d^
**TTS-1**	Template-P1	Py – TFO1	22 ±1	-50 ±10
		Pu – TFO4	190 ±2	-20 ±10
	Sense-P2	Py – TFO1	20 ±2	+70 ±10
		Pu – TFO4	13 ±1	-80 ±10
**TTS-2**	Template-P3	Py – TFO1	8 ±2	+40 ±10
		Pu – TFO4	7 ±1	+70 ±10
	Sense-P4	Py – TFO1	21 ±1	+70 ±10
		Pu – TFO4	148 ±2	-30 ±10
**TTS-3**	P2 + P4	Pu – TFO4	63 ±1	-90 ±10
		Py – TFO1	120 ±1	+210 ±20
	P1 + P4	Pu – TFO4	12 ±1	-70 ±10

^a^Template indicates the homopurine sequence is in the template strand, while sense indicates the homopurine sequence is in the sense strand; ^b^Py and Pu indicates a pyrimidine- or purine-motif triplex, respectively; ^c^calculated concentration of the TFO that exerts half-maximal effect on the transcription rate of Broccoli; ^d^ the maximal modulation effect was calculated as the normalized transcription rate at the right asymptote of the sigmoid (for additional details, see Supplementary data).

Based upon a number of experimental and theoretical studies, the hypothesis that natural triplex structures can modulate transcription with both inhibitory and enhancing effects is not farfetched (14,17–19,32). In order to explore this idea further, different promoter designs were tested. Our central question dealt with how shifting the TTS to other positions would influence transcription. Thus, in the first instance, the 15 base polypurine sequence was moved to the sense strand, again in the gap between the consensus –35 and –10 sites, termed promoter P2. Furthermore, a second position was considered (TTS-2), which was located immediately downstream the -10 consensus sequence on either the antisense or sense strand (P3 containing the polypurine in the template strand and P4 containing the polypurine in the sense strand). The three promoters were tested under the same conditions used for P1 (*vide supra*). Again, fully in harmony with the EMSA assay, results indicate that the effects on the Broccoli transcription rate depend on triplex formation and was found to be significant for TFOs 1 and 4, Figure 3, panels C and D, while it was negligible in the case of the mixed motif TFOs, Supplementary Figures S3, S4 and S5. Intriguingly, moving TTS-1 from the template to the sense strand, a 70% enhancement of the transcriptional activity was observed upon incubation with TFO1, whereas TFO4 displayed an 80% inhibition (Table 1).

**Figure 4.**
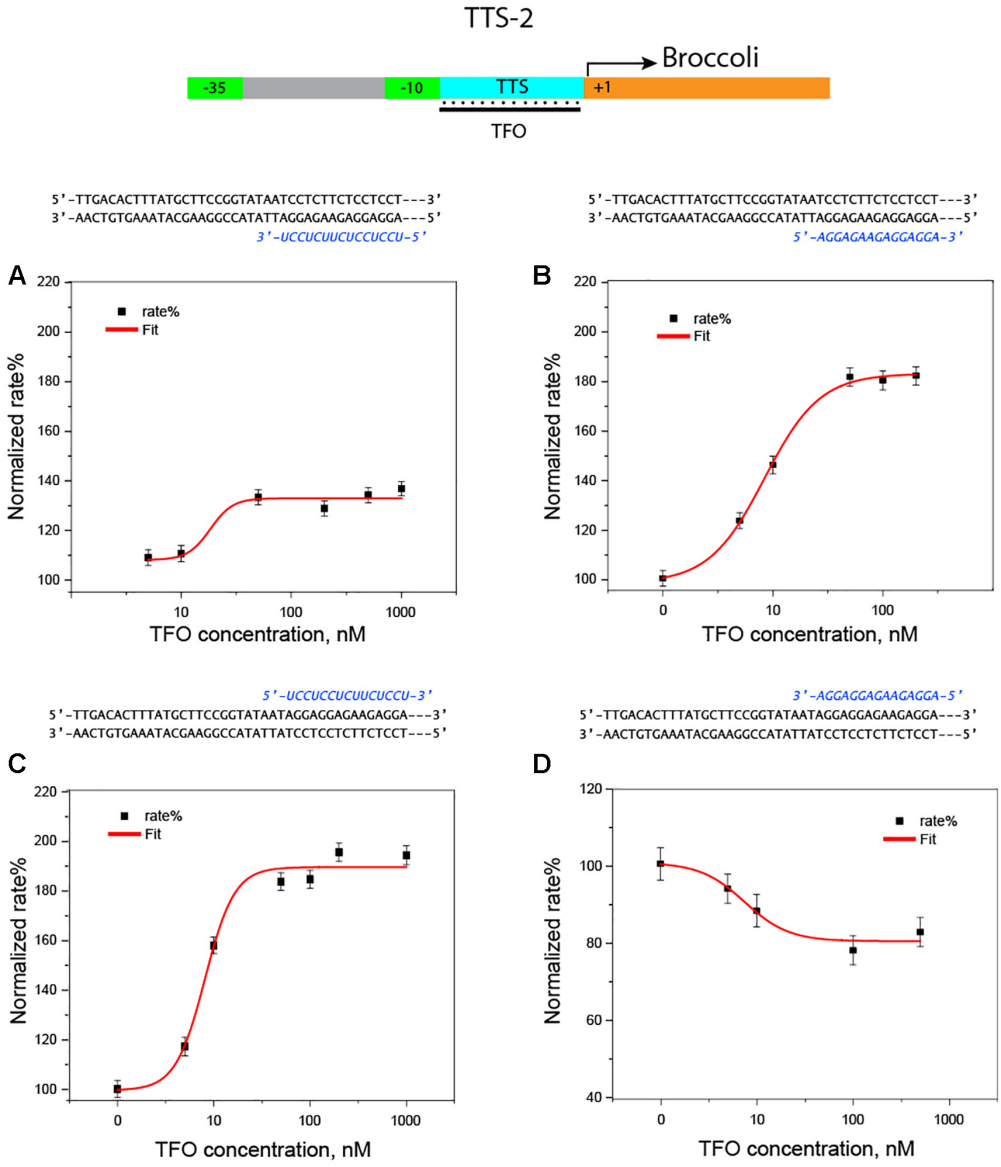
Analysis of the triplex formation effect on the production of Broccoli from transcription units engineered to contain TTS-2, as described in Figure 1C. (**A** and **B**) Kinetic analysis of the transcription unit containing TTS-2, with the polypurine sequence in the template strand (P3), in the presence of different concentrations of TFO1 and 4, respectively. (**C** and **D**) Kinetic analysis of the transcription unit containing TTS-2, with the polypurine sequence in the sense strand (P4), in the presence of different concentrations of TFO1 and 4, respectively. The three dashes (—) indicate the same duplex portion for all designs, namely, Broccoli template.

The results for the promoters containing the polypurine sequences downstream of the –10 domain in the template (P3) or sense strand (P4) are displayed in Figure 4. Noteworthy, when TTS-2 was placed in the template strand, an enhancement of the transcriptional activity was registered both with TFO1 and 4, Figure 4, panels A and B, which is the opposite behavior with respect to TTS-1 in P1. By contrast, the effect of placing TTS-2 at the sense strand was similar to the one observed for TTS-1. TFO1 led to an enhancement of RNA production superimposable to the one registered for TTS-1, while TFO4 inhibited Broccoli transcription, as depicted in Figure 4, panels C and D, respectively (for the results using mixed motif TFOs, see Supplementary data, Figures S4 and S5).

Summarizing, when TTS-1 was located at the template strand, which represents the model target for inhibition, both TFO1 and 4 hindered transcription as expected. In addition, the inhibitory effect follows the stability of the binding. The situation was inverted when TTS-2 was located on the template strand, in fact both TFO1 and TFO4 enhanced the RNA production. Differently, when TTS-1 and 2 were located at the sense strand, TFO1 enhanced the transcriptional activity whereas TFO4 displayed inhibition. Thus, both TTS positions and TFO1 and 4 can be suitably designed to up- or downregulate transcription of the fluorescent aptamer.

Interestingly, the relationship between *K*_d_ values measured by EMSA and the EC_50_ values observed in the polymerization experiments seems to be relevant only when inhibition of transcription is involved. In fact, the phenomenon of inhibition was attributed to a competition mechanism of the RNAp and the TFO for the same binding site within the promoter region (9–12). Differently from inhibition, the unprecedented transcription enhancement, reported here for the first time, seems to follow a non-trivial mechanism which warrants further investigation.

### *Broccoli* production controlled by promoters containing two TTSs

Besides the unprecedented enhancement of transcriptional activity, the ability to modulate RNA production via triplex formation is noteworthy in view of potential applications in synthetic gene circuits. As a first step in this direction, the modularity of the platform was studied by increasing the number of binding sites within the same promoter and by increasing the number of binding sequences in the same TFO.

Firstly, TTS-1 and TTS-2 were modularly combined into engineered promoters that contained two triplex target sites (TTS-3) as depicted in Figure 1C. The production of fluorescent aptamer from TTS-3 was assessed in the presence of TFO1 or 4. Secondly, the effect of triplex formation was characterized in the context of a simple serial circuit, in which a regulatory TFO was produced from one transcription template to control transcription of Broccoli from another. Lastly, transcription units were designed to produce TFOs that contained two TFO4 motifs in series, separated by a spacer containing 4, 5, 6, 7 or 8 nucleotides (TFO5, 6, 7, 8 and 9); see Supplementary data, Table S1, for the sequence design. Thus, the transcriptional activities of TTS-3 (*vide infra*) were determined in the presence of TFOs 5–9.

Ideally, for a single TFO (i.e. TFO1 or TFO4), eight regulatory regions of type TTS-3 can be constructed by simple combinations, where two strands comprising the same TFO sequence bind simultaneously within the promoter and in the downstream region (for details and a complete scheme of all possible combinations, see Supplementary data, Scheme S1). Furthermore, to maximize the observed effect, only configurations that form triplex structures with mutual enhancement or inhibitory effects were selected. Thus, four potential TTS-3 combinations were designed for hosting TFO1 or 4, respectively.

**Figure 5.**
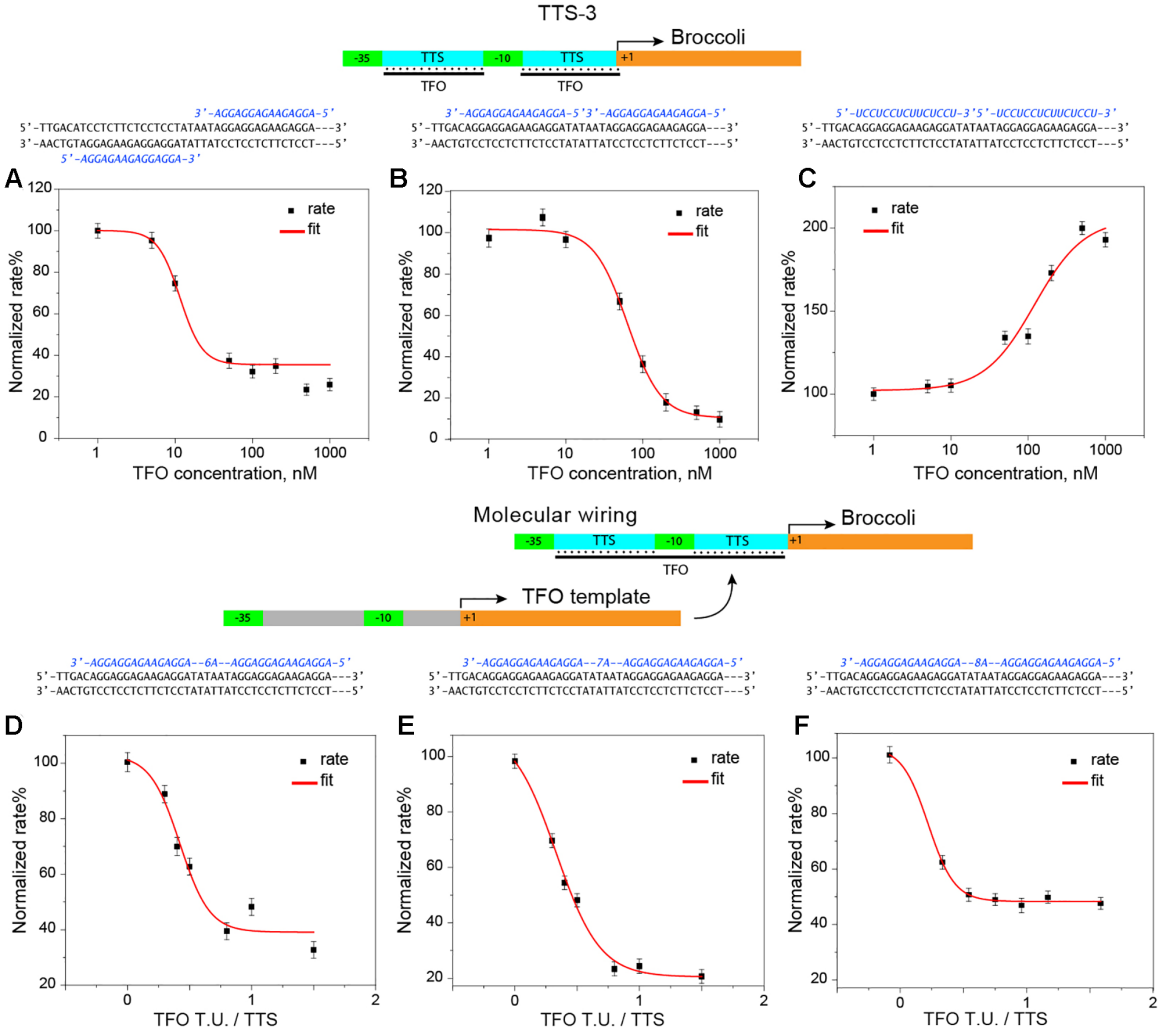
Analysis of the triplex formation effect on the production of Broccoli fom transcription units engineered to contain TTS-3, as described in Figure 1C. (**A** and **B**) Effect of different concentrations of TFO4 on RNA polymerization from promoter P1 + P4 and P2 + P4. (**C**) Effect of different concentrations of TFO1 on the RNA polymerization from promoter P2 + P4. (**D**–**F**) Effect of different amounts of TFO4-producing transcription unit (i.e. TFO T.U./TTS ratio) on the RNA polymerization from promoter P2 + P4, with TFOs containing spacers equal to 6, 7 and 8 bases, respectively (for TFOs containing 4- and 5-base spacers, see SI). The three dashes (—) indicate the same duplex portion for all designs, namely, Broccoli template.

The experimental results show that engineered TTS-3 promoters that exerted an effect on transcription were P2 + P4 for TFO1 while P1 + P4 and P2 + P4 showed regulation in the presence of TFO4. These promoters were subjected to increasing concentrations of the corresponding TFO as previously described for promoters with a single triplex target site. Figure 5, panels A and B, shows the TFO4-dependent normalized rates of Broccoli production from promoters P1 + P4 and P2 + P4, respectively. The TFO-dependent rates were fitted with sigmoidal functions, from which the EC_50_ values were calculated to be 12 ± 1nM and 63 ± 1nM, while the transcriptional activity was inhibited by 70% and 90%, respectively. Similarly, Figure 5, panel C, shows the analysis of the TFO1-dependent normalized rates of Broccoli transcription from promoter P2 + P4, for which a sigmoidal fit resulted in an EC_50_ of 120 ± 1 nM. Noteworthy, the transcription was 2-fold enhanced for this combination (i.e. 210%) and it was the highest enhancement obtained in the present study, indicating that the enhancement effect is strengthened by the presence of both TTSs in the sense strand. Table 1 summarizes the experimental parameters obtained for TTS-3s.

In general, the shift of EC_50_ to higher values suggests a decrease in the affinity of the TFOs for their target site on the promoter. Notably, the effect on TTS-3 ranged from a doubling to almost silencing the transcriptional activity of the promoter, showing the tunability of the system which allows broad tuning of gene circuit parameters.

### Molecular wiring of engineered promoters

In biological systems, transcription units belonging to different genomic regions can communicate and operate logic functions by their interaction with freely diffusing short nucleic acids and proteins acting as wiring molecules. Herein, to evaluate the effect of molecular wiring on the engineered P2 + P4 promoter, TFO4 or an extended TFO4 were directly produced *in situ* by a system involving two transcription units. Transcription units were designed to contain the template for two purine-motif containing TFOs, separated by a spacer 4- to 8-nucleotides long. The kinetic analysis of Broccoli production from promoter P2 + P4 in the presence of increasing excesses of TFO transcription unit showed an inhibitory effect of 80% with an EC_50_ = 12 nM for two-tandem repeats (i.e. at a 0.4:1 ratio of TFO to Broccoli transcription unit). Figure 5D–F show the respective experimental results for spacers 6, 7, and 8. Comparing the EC_50_ values and maximum effects, clearly, only a minute difference is detected among the different spacer length (for the kinetic analysis of the molecular wiring comprising the 4- and 5-nucleotide long spacer TFO, see Supplementary data, Figure S6).

With respect to P2 + P4, the transcription units for the TFOs were used in the following concentration ratios: 0:1, 0.5:1, 0.75:1, 1:1, 1.25:1, 1.5:1, 1.75:1, 2:1, namely at the ratio 1:1 the TFO-transcription unit and P2 + P4 were added to the mixture at the same concentration. Moreover, to compensate for the increase of the total promoter concentration and account for resource sharing effects of the RNAp, a third transcription unit, producing a polyadenine (polyA) RNA strand, was introduced into the system. The results showed the feasibility of the *in situ* produced TFO to exert an effect on a secondary transcription unit containing the TTS (i.e. the Broccoli transcription unit).

## DISCUSSION

It is known that the formation of a triplex structure at the promoter of a transcription unit inhibits RNA polymerization by RNAp. As a general concept, this effect can be ascribed to competitive binding of the TFO and the RNAp to the promoter, which reflects the quantitative relationship between the stability of the hybrid triplex in comparison to the transcription initiation complex.

In the present study, a proof of concept showing, besides inhibition, the modulation of RNA transcription upon nucleic acid triplex formation, as an ON/OFF switch, was presented. Firstly, as a reference model, a system was designed to reproduce the already reported phenomenon of inhibition using the general architecture of the *E. coli* promoter (25). As expected, inhibition took place when pyrimidine or purine triplexes were formed on TTSs containing the polypurine sequence in the region between the -35 and -10 domains of the template strand (P1), namely in between the RNA polymerase binding sites. Moreover, transcription enhancement is here reported for the first time, an effect which is likely associated with the different geometries of the pyrimidine motif (TFO parallel to the polypurine sequence) and the purine motif (TFO antiparallel to the polypurine sequence). This enhancement of transcription can not be described by a simple competition mechanism based on triplex strength, as the comparison between the EC_50_ values reported in Table 1 and the *K*_d_ values show, and it will require the screening of a properly sized library comprising different TTSs and TFOs.

As already suggested, the formation of a triplex near a promoter induces a distortion in the duplex DNA that allows the RNAp to interact with the single-stranded template more easily and thus to initiate transcription at a higher rate (33,34). This is in agreement with the results of the TTS-3 design, where the modular combination of two enhancing triplexes at the promoter does not further increase the enhancement effect. On the other hand, the modular combination of two inhibitory triplexes further increases the inhibition effect due to the greater binding competition with RNAp, supporting the view that different mechanisms are at play for enhancing or inhibiting triplexes.

In addition to the effects of added synthetic TFOs, to demonstrate the feasibility to generate transcription unit circuits, we showed that the promoter containing the TTS downstream the –10 domain exhibited a similar behavior when the polypurine sequence was placed in the template strand. Enhancement of transcription was observed when pyrimidine or purine triplexes were formed. When the polypurine sequence was placed in the sense strand, symmetric effects as for P2 were observed for the pyrimidine (enhancement) or purine (inhibition) triplexes, Figure 4.

Importantly, TFO-producing transcription units were able to affect the transcription from engineered downstream promoters. This showed the possibility to wire the different transcription units with inhibitory effect. Also, due to the geometry of the triplex, the spacer sequence separating the two TFO domains could, in principle, be further engineered to contain an additional regulatory sequence (e.g. an aptamer) to effectively turn the sequence into a riboswitch that would form the triplex only in the presence of the stimulus or ligand.

In conclusion, we demonstrated the possibility to build engineered transcription units, which were properly regulated by hybrid RNA–DNA triplex structures formed at the promoter. While such a modulation mechanism is similar to protein-mediated transcription regulation, an RNA-only regulatory machinery based on triplex structure formation is unprecedented, especially for transcription rate enhancement (32). Most importantly, whether the effects of the triplex structures will be inhibitory or enhancing can be rationally designed by the choice of the triplex motif (pyrimidine or purine), the position of the polypurine sequence (sense or template strand), and the location of the triplex with respect to the conserved sequences of the promoter. The proper combination of these factors can be used to set ON or OFF the transcription of a target promoter. Furthermore, the modularity of the strategy allows the design of promoters with multiple triplex target sites characterized by a stronger inhibitory effect. The use of TFO producing transcription units allowed wiring of the system, indeed showing the possibility to use this strategy to add an additional RNA-only layer of control for building transcription circuits and nucleic acid-based logic gates (35), and aiming at introducing it *in vivo* as a new bio-orthogonal transcription modulation approach.

## DATA AVAILABILITY

The data underlying this article are available in the article and in its online supplementary material.

## Supplementary Material

gkac1131_Supplemental_File
