# Supplementary material for: Rational design of hybrid DNA–RNA triplex structures as modulators of transcriptional activity in vitro
Source: Nucleic Acids Res. 2022 Dec 20;50(22):13172–82. doi: 10.1093/nar/gkac1131 (PMC9825147; doi:10.1093/nar/gkac1131)
Supplement: gkac1131_Supplemental_File [file gkac1131_supplemental_file.pdf]

Rational design of hybrid DNA-RNA triplex structures as modulators of transcriptional activity *in vitro*

Alessandro Cecconello,* Massimiliano Magro, Fabio Vianello, Friedrich C. Simmel*

Table of Contents

Materials 2
Experimental Procedures 3
Results and discussion 4
Author Contributions 8

Materials

All DNA and RNA sequences were ordered from IDT (Integrated DNA Technologies, USA) as freeze-dried samples. Sequences are detailed in Table S10. Solutions were prepared in ultrapure water (resistivity at least 18MOhm cm) using a Sartorius Arium Pro Ultrapure Lab Water System. KCl, NaCl, Tris-acetate-EDTA (TAE), N,N,N',N'-tetramethylethylenediamine (TEMED), and Tris were purchased from ROTH. Magnesium acetate was purchased from Fluka. SybrGold nucleic acid stain, Triton X-100, and ammonium persulfate were purchased from ThermoScientific. Acrylamide/bis-acrylamide solution was purchased from Sigma Aldrich/Merck. Broccoli fluorescent ligand (Z)-4-(3,5-difluoro-4-hydroxybenzylidene)-2-methyl-1-(2,2,2-trifluoroethyl)-1H-imidazol-5(4H)-one (DFHBI-1T) was purchased from Lucerna. σ70-saturated *E. coli* RNA polymerase (holoenzyme) was purchased from New England Biolabs (NEB). Dithiothreitol was purchased from Biochemica.

Double strand DNA annealing was carried out using a Mastercycler Nexus-GX from Eppendorf. All RNA polymerization experiments were carried out using a Clariostar plate reader from BMG labtech with samples loaded in 96-well plates from Corning.

Table S1. DNA sequences used in this work are listed 5' to 3'. TU= transcription unit.

Name	Sequence
TTS1	TCCTCTTCTCCTCCT
TTS2	AGGAGGAGAAGAGGA
P1(sense)	TTGACATCCTCTTCTCCTCCTATAATAAAAAAA<br>GGAACGAGACGGTCGGGTCCAGATATTCGTATCTGTCGAGTAGAGTGTGGGCTCGTTCC
P1(template)	GGAACGAGCCCACACTCTACTCGACAGATACGAATATCTGGACCCGACCGTCTCGTTCC<br>TTTTTTTATTATAGGAGGAGAAGAGGATGTCAA
P2(sense)	TTGACAGGAGGAGAAGAGGATATAATAAAAAAA<br>GGAACGAGACGGTCGGGTCCAGATATTCGTATCTGTCGAGTAGAGTGTGGGCTCGTTCC
P2(template)	GGAACGAGCCCACACTCTACTCGACAGATACGAATATCTGGACCCGACCGTCTCGTTCC<br>TTTTTTTATTATAGGAGGAGAAGAGGATGTCAA
P3(sense)	TTGACACTTTATGCTTCCGGTATAATCCTCTTCTCCTCCT<br>GGAACGAGACGGTCGGGTCCAGATATTCGTATCTGTCGAGTAGAGTGTGGGCTCGTTCC
P3(template)	GGAACGAGCCCACACTCTACTCGACAGATACGAATATCTGGACCCGACCGTCTCGTTCCAGGAGGAGAAGAGG<br>ATTATACCGGAAGCATAAAGTGTCAA
P4(sense)	TTGACACTTTATGCTTCCGGTATAATAGGAGGAGAAGAGGA<br>GGAACGAGACGGTCGGGTCCAGATATTCGTATCTGTCGAGTAGAGTGTGGGCTCGTTCC
P4(template)	GGAACGAGCCCACACTCTACTCGACAGATACGAATATCTGGA<br>CCCGACCGTCTCGTTCCTCCTCTTCTCCTCCTATTATACCGGAAGCATAAAGTGTCAA
P1+P4(sense)	TTGACATCCTCTTCTCCTCCTATAAT<br>AGGAGGAGAAGAGGA GGAACGAGACGGTCGGGTCCAGATATTCGTATCTGTCGAGTAGAGTGTGGGCTCGTTCC
P1+P4(template)	GGAACGAGCCCACACTCTACTCGACAGATACGAATATCTGGACCCGACCGTCTCGTTCCTCCTCTTCTCCTCCT<br>ATTATAGGAGGAGAAGAGGATGTCAA
P2+P4(sense)	TTGACAGGAGGAGAAGAGGATATAAT<br>AGGAGGAGAAGAGGA GGAACGAGACGGTCGGGTCCAGATATTCGTATCTGTCGAGTAGAGTGTGGGCTCGTTCC
P2+P4(template)	GGAACGAGCCCACACTCTACTCGACAGATACGAATATCTGGACCCGACCGTCTCGTTCCTCCTCTTCTCCTCCT<br>ATTATATCCTCTTCTCCTCCTGTCAA
polyA TU(sense)	TTGACACTTTATGCTTCCGGTATAAT<br>AAAAAAATAAAAATAAAAATAAAAATAAAAATAAAAATAAAAATAAAAATAAAAATAAAAAT

polyA TU(template)	ATTTTTATTTTTATTTTTATTTTTATTTTTATTTTTATTTTTATTTTTATTTTTATTTTTTT ATTATACCGGAAGCATAAAGTGTCAA
2XTFO4 spacer-4TU (sense)	TTGACACTTTATGCTTCCGGTATAATAAAAAAAAAGGAGAAGAGGAGGAAAAAAGGAGAAGAGGAGGA
2XTFO4 spacer-4TU (template)	TCCTCCTCTTCTCCTTTTTTCCTCCTCTTCTCCTTTTTTTTATTATACCGGAAGCATAAAGTGTCAA
2XTFO4 spacer-5TU (sense)	TTGACACTTTATGCTTCCGGTATAATAAAAAAAAAGGAGAAGAGGAGGAAAAAAAGGAGAAGAGGAGGA
2XTFO4 spacer-5TU (template)	TCCTCCTCTTCTCCTTTTTTTCCTCCTCTTCTCCTTTTTTTTATTATACCGGAAGCATAAAGTGTCAA
2XTFO4 spacer-6TU (sense)	TTGACACTTTATGCTTCCGGTATAATAAAAAAAAAGGAGAAGAGGAGGAAAAAAAAGGAGAAGAGGAGGA
2XTFO4 spacer-6TU (template)	TCCTCCTCTTCTCCTTTTTTTTCCTCCTCTTCTCCTTTTTTTTATTATACCGGAAGCATAAAGTGTCAA
2XTFO4 spacer-7TU (sense)	TTGACACTTTATGCTTCCGGTATAATAAAAAAAAAGGAGAAGAGGAGGAAAAAAAAAGGAGAAGAGGAGGA
2XTFO4 spacer-7TU (template)	TCCTCCTCTTCTCCTTTTTTTTTCCTCCTCTTCTCCTTTTTTTTATTATACCGGAAGCATAAAGTGTCAA
2XTFO4 spacer-8TU (sense)	TTGACACTTTATGCTTCCGGTATAATAAAAAAAAAGGAGAAGAGGAGGAAAAAAAAAAGGAGAAGAGGAGGA
2XTFO4 spacer-8TU (template)	TCCTCCTCTTCTCCTTTTTTTTTTCCTCCTCTTCTCCTTTTTTTTATTATACCGGAAGCATAAAGTGTCAA

Experimental Procedures

Double strand DNA structures were produced by annealing the strands in a temperature ramp (90°C to 4°C) in 1 hour in the Mastercycler, in an aqueous solution containing 10µM of each strand and 50mM KCl. dsDNA stock solutions were stored at -20°C and used when needed at the appropriate final concentration.

EMSA experiments were carried out in the recommended NEB buffer for RNA polymerization, i.e., 40Mm Tris-HCl, 150mM KCl, 10mM $MgCl_2$, 1mm DTT, 0.01% Triton X-100, at pH 7.5. Samples contained a constant TTS concentration equal to 100nM and increasing concentrations of the specific TFO in the interval 0-10µM for TFOs 1, 3, and 4 while for TFO2 the interval of concentrations was 0-100µM. Although for higher TFO concentrations saturation of the gel pores was unavoidable, to mitigate such effect samples were diluted 1:2 prior to loading into the wells. MgAcetate was added to reach a final concentration of Mg^{2+} (NEB buffer+MgAcetate) equal to 30mM. 10% acrylamide/bis-acrylamide 19:1 gel was prepared following an established procedure in TAE 1x buffer, containing 10mM $MgCl_2$. The gel was run at 50V 10mA for 2 hours in an ice bath and then stained with SybrGold. Gel pictures were acquired using a gel imager from ThermoScientific. Bands were analyzed using Fiji software and the band intensities were fitted using Origin Lab software. Errors in the band quantification where estimated comparing adjacent band with similar intensities and were introduced into the plots as error bars. The K_d values were obtained from three independent experiments and were estimated using standard assumptions: (a) no ssDNA is present due to high ionic strength (10mM Mg^{2+} in the gel buffer and the running buffer) and low temperature (the runs where performed in an ice bath); (b) when the duplex band was not visible, the maximum amount of triplex (dsTTS+ssTFO) was formed; (c) when the triplex band is not visible, no triplex is formed and all duplex TTS is free; and (d) when the triplex and the dsTTS have the same concentration, the following definition of K_d can be used.

$$\mathrm{Kd} = \frac{[\mathrm{dsTTS}] \cdot [\mathrm{ssTFO}]}{[\mathrm{Triplex}]} \qquad \text{when } [\mathrm{dsTTS}] = [\mathrm{Triplex}] \qquad \text{then } \mathrm{Kd} = [\mathrm{ssTFO}]$$

In order to estimate the ssTFO concentration for which the above condition is true, either the duplex band relative intensities or the triplex band relative intensities where plotted against the respective ssTFO concentration, and the experimental data were fitted with a

sigmoid. The ssTFO concentration corresponding to the sigmoid symmetry point was used as K_d value. Similar methods were used in previous studies.(1, 2)

Melting curves experiments were conducted using the same buffer described in the previous paragraph. The solution containing 100nM of the double strand TTS and 1µM of the selected TFO was annealed at 4°C for 30 minutes, then poured in a quartz cuvette and topped with few drops of decane ($C_{10}H_{22}$) to prevent evaporation. Melting curves were then analyzed using Origin Lab software.

RNA polymerization experiments using purified TFOs were carried out using a plate reader where each sample was prepared in 25 µL final volume. Samples were prepared 30' in advance while triplex formation was conducted at room temperature for 30 minutes, in buffered solutions containing excess of rNTPs, 20nM transcription unit, and the TFO at the specific concentration. Immediately before starting collection of fluorescence, 50 µM DFHBI-1T and 0.1U RNA polymerase holoenzyme were added to the samples and the plate was covered with a transparent seal. The plate was then loaded in the plate reader set at 30°C and a 5 hours fluorescence signal collection was started, with excitation 420-470nm and fluorescence signal recoded at 515-520nm. Time-dependent fluorescence signals were then analyzed using Origin Lab software where the rates for each sample (y) were fitted linearly and then plotted against TFO concentration (x). The experimental points were the fitted using a dose-response equation:

$$y = A1 + \frac{A2 - A1}{1 + 10^{(logx_0 - x)p}}$$

Where A1 and A2 are the left and right asymptotes, respectively, x_0 is the TFO concentration at the inflection point (i.e., E_{50}), and p is the slope of the sigmoid at the inflection point.

Experiments involving co-transcriptional formation of the triplex structures were carried out without initial incubation at room temperature. 25 µL samples were assembled in the same buffer described earlier, containing the three transcription units (i.e., Broccoli transcription unit, TFO transcription unit, and polyA transcription unit). The transcription unit total concentration, for each sample, was kept constant at 150nM.

Results and discussion

Electrophoresis experiment results, Figure S1, show the appearance of one or two new bands for samples containing increasing concentrations of TFO. While for mixed motifs TFO2 and TFO3 the target DNA duplex does not change intensity, this was used to evaluate the efficiency of the triplex formation. Conversely, for TFO1 and TFO4, the appearance of a new band at higher molecular weight corresponds to the disappearance of the TTS. For this reason, the band corresponding to the triplex was used to evaluate the efficiency of the triplex formation. TFO4 gel shows three bands at TFO concentrations around 1µM. This is due to the lower stability of the triplex and the higher amount of TFO needed to generate the triplex, which resulted in an excess of TFO accumulating in the solution and appearing as a third band.

The linear rates of the RNA polymerization experiments were fitted in a time interval of two hours, starting after 1 hour from the beginning of the fluorescence signal collection. During the first hour the fluorescence signal was erratic, probably due to the inhomogeneous temperature of the plate, and those points were not used. Similarly, the linear kinetics started to get slower after several hours from the beginning of the experiments resulting in an unreliable linear fitting. For these reasons an interval of two hours was chosen. Figure s1, panels A, B, C, and D show representative kinetics for transcription unit containing a single TTS (P1) in the presence of TFO1, TFO2, TFO3, or TFO4 in the concentration interval 0-1µM, respectively. Curves where translated vertically to improve clarity.

Co-transcriptional triplex formation experiments were carried out in the presence of three transcription units. A transcription unit containing the template for Broccoli aptamer, a transcription unit containing the template for the selected TFO, and a transcription unit containing the template for a ssRNA that was not interacting with dsDNA (i.e., an adenine-rich oligonucleotide, polyA). In addition, the specific transcription unit for polyA or polyT was selected in order to avoid formation of double strand RNA structures with the TFO. The amount of each of the three transcription units was calculated to result in a constant total amount of transcription units in each sample.

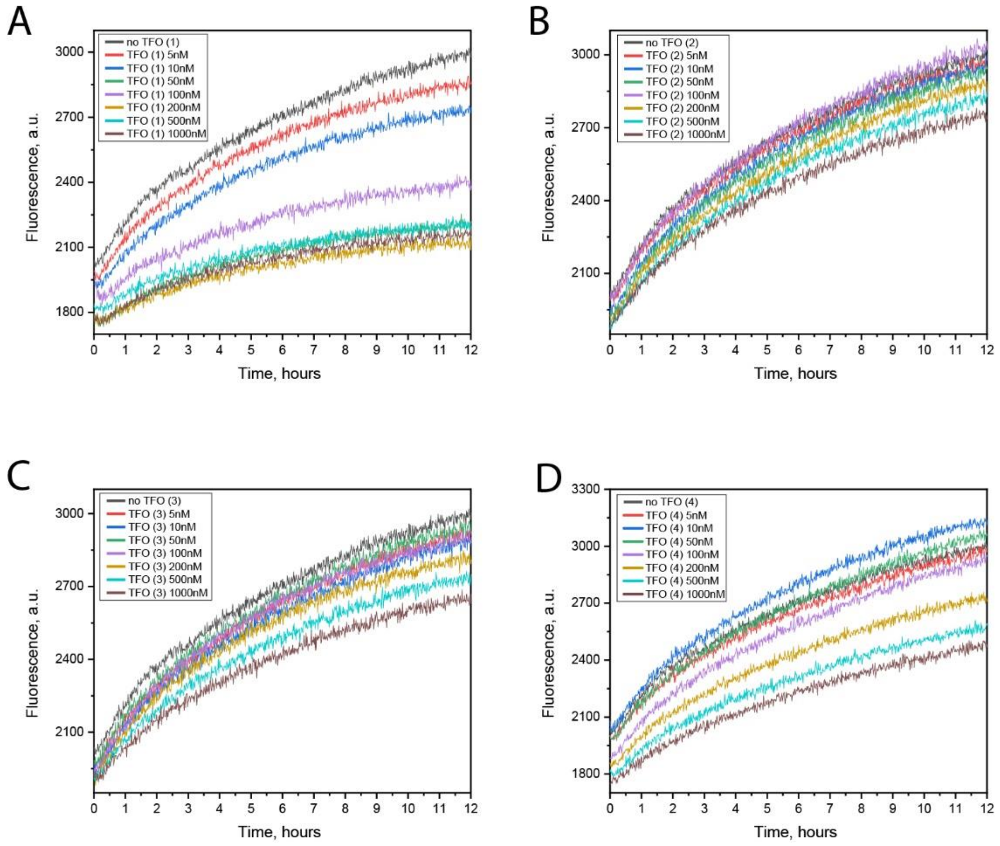

Figure S1. *In vitro* Broccoli biosynthesis kinetics from transcription unit P1 containing one TTS between positions -35 and -10 followed by the time-dependant fluorescence changes in the presence of: Panel A – increasing amounts of TFO1; panel B – increasing amounts of TFO2; panel C – increasing amounts of TFO3; and panel D – increasing amounts of TFO4.

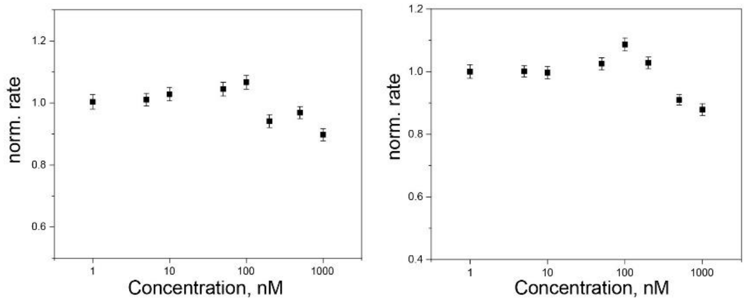

Figure S2. Analysis of the triplex formation effect on the production of Broccoli from transcription units engineered to contain TTS-1, as described in Figure 1C. Kinetic analysis of the transcription unit containing TTS-1, with the polypurine sequence in the template strand (P1), in the presence of different concentrations of mixed motif TFOs 2 or 3, left panel and right panel, respectively.

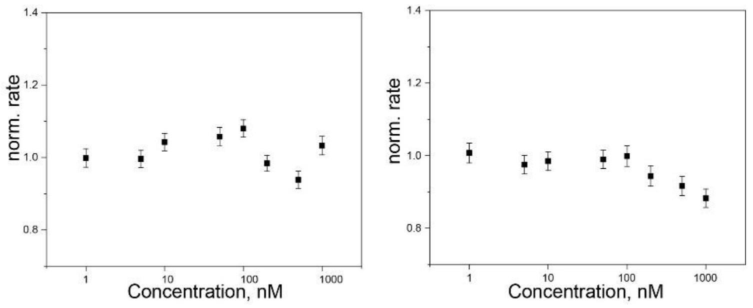

Figure S3. Analysis of the triplex formation effect on the production of Broccoli from transcription units engineered to contain TTS-1, as described in Figure 1C. Kinetic analysis of the transcription unit containing TTS-1, with the polypurine sequence in the sense strand (P2), in the presence of different concentrations of mixed motif TFOs 2 or 3, left panel and right panel, respectively.

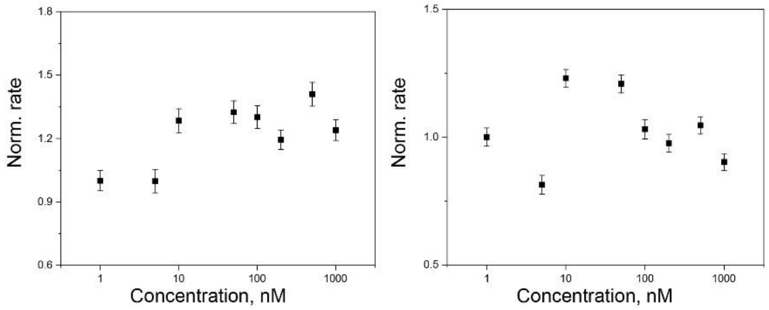

Figure S4. Analysis of the triplex formation effect on the production of Broccoli from transcription units engineered to contain TTS-2, as described in Figure 1C. Kinetic analysis of the transcription unit containing TTS-2, with the polypurine sequence in the template strand (P3), in the presence of different concentrations of mixed motif TFOs 2 or 3, left panel and right panel, respectively.

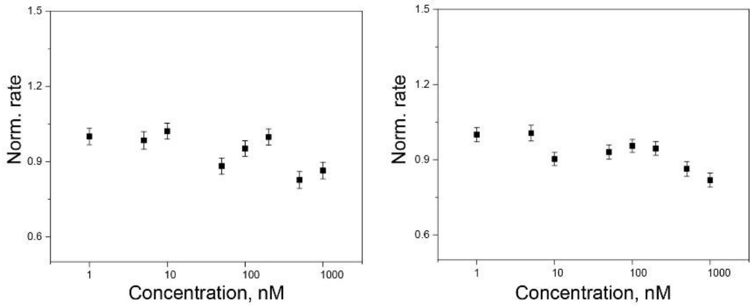

Figure S5. Analysis of the triplex formation effect on the production of Broccoli from transcription units engineered to contain TTS-2, as described in Figure 1C. Kinetic analysis of the transcription unit containing TTS-2, with the polypurine sequence in the sense strand (P4), in the presence of different concentrations of mixed motif TFOs 2 or 3, left panel and right panel, respectively.

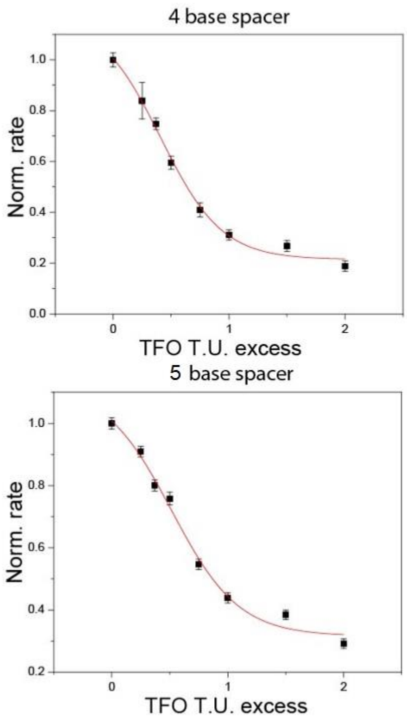

Figure S6. Analysis of the effect of increasing relative excesses of a transcription unit for the polymerization of an RNA containing 2 TFO4 sequences separated by a spacer containing 4 bases (top panel) or 5 bases (lower panel), on a target promoter P2+P4.

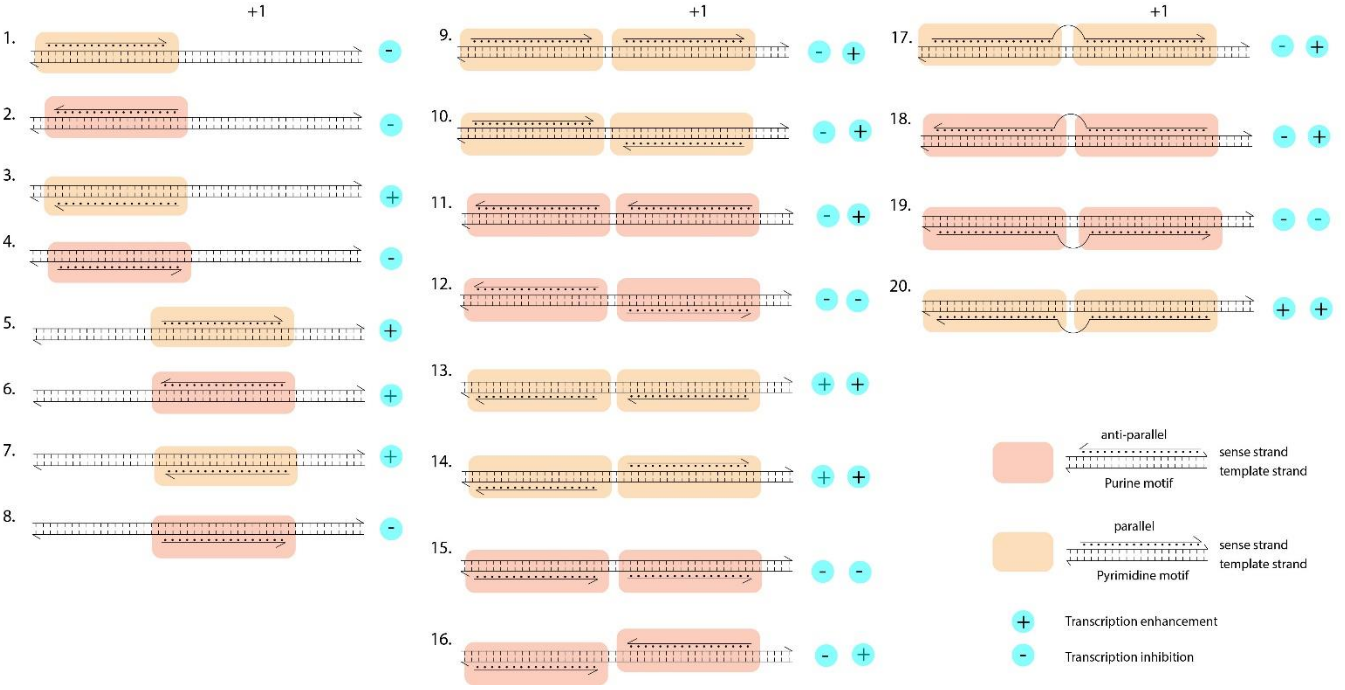

Scheme S1. Triplex configurations with engineered promoters. Schemes 1-8: Single TTS containing promoters where the TTS is placed between the two domains -35 and -10, schemes 1-4 (i.e., TTS-1), upstream of the polymerization starting point +1, or where the TTS is placed downstream the -10 domain, schemes 5-8 (i.e., TTS-2). Schemes 9-16: Combinations of two TTSs, i.e., TTS-3. Schemes 17-20: TTS-3 where the RNA comprises two TFO domains connected via a spacer sequence. Plus and minus signs indicate the experimental enhancement (+) or inhibition (-) effect of the triplex formation in respect to transcription of Broccoli aptamer.

Author Contributions

A.C. and F.C.S. formulated the project and designed the experiments. A.C. performed the experiments. A.C., F.C.S., M.M., and F.V. wrote the paper.

Reference

1. Kotkowiak,W., Kotkowiak,M., Kierzek,R. and Pasternak,A. (2014) Unlocked nucleic acids: implications of increased conformational flexibility for RNA/DNA triplex formation. *Biochem. J*, **464**, 203–211.

2. Kunkler,C.N., Hulewicz,J.P., Hickman,S.C., Wang,M.C., McCown,P.J. and Brown,J.A. (2019) Stability of an RNA•DNA–DNA triple helix depends on base triplet composition and length of the RNA third strand. *Nucleic Acids Res.*, **47**, 7213–7222.
